# The genetic diversity, replication, and transmission of 2009 pandemic H1N1 viruses in China

**DOI:** 10.3389/fmicb.2023.1110100

**Published:** 2023-02-17

**Authors:** Hailiang Sun, Yongcui Wang, Hanlin Liu, Zifeng Pang, Xinxin Cui, Rui Zhao, Yanwei Liu, Xiaoyun Qu, Mian Huang, Changwen Ke, Ming Liao

**Affiliations:** ^1^College of Veterinary Medicine, South China Agricultural University, Guangzhou, China; ^2^National and Regional Joint Engineering Laboratory for Medicament of Zoonosis Prevention and Control, South China Agricultural University, Guangzhou, China; ^3^Key Laboratory of Zoonosis Control and Prevention of Guangdong Province, South China Agricultural University, Guangzhou, China; ^4^The Northwest Institute of Plateau Biology, Chinese Academy of Sciences, Xining, China; ^5^Guangzhou Zoo, The People’s Government of Guangzhou Municipality, Guangzhou, China; ^6^Guangdong Provincial Center for Disease Control and Prevention, The People’s Government of Guangzhou Municipality, Guangzhou, China

**Keywords:** influenza, haplotypes analysis, replication, transmission, phylogenetic analysis

## Abstract

**Background:**

The 2009 pandemic H1N1 influenza A virus (pdm09) continue to evolve, and few studies have systemically analyzed the evolution, replication, and transmission of pmd09 viruses in China.

**Methods:**

To better understand the evolution and pathogenicity of pdm09 viruses, we systematically analyzed viruses that were confirmed in 2009–2020 in China and characterized their replication and transmission ability. We extensively analyzed the evolution characteristics of pdm/09 in China over the past decades. The replication ability of 6B.1 and 6B.2 lineages on Madin-Darby canine kidney (MDCK) and human lung adenocarcinoma epithelial (A549) cells and their pathogenicity and transmission in guinea pigs were also compared.

**Results:**

In total, 3,038 pdm09 viruses belonged to clade 6B.1 (62% of all pdm09 viruses) and clade 6B.2 (4%). Clade 6B.1 pdm09 viruses are the predominant clade, with proportions of 54.1%, 78.9%, 57.2%, 58.6%, 61.7%, 76.3%, and 66.6% in the North, Northeast, East, Central, South, Southwest, and Northeast regions in China, respectively. The isolation proportion of clade 6B.1 pdm/09 viruses was 57.1%, 74.3%, 96.1%, 98.2%, 86.7%, and 78.5% in 2015–2020, respectively. A clear differentiation time point appeared in 2015 before which the evolution trend of pdm09 viruses in China was similar to that in North America but then showed a different trend after that point. To characterize pdm09 viruses in China after 2015, we further analyzed 33 pdm09 viruses isolated in Guangdong in 2016–2017, among which A/ Guangdong/33/2016 and A/Guangdong/184/2016 (184/2016) belonged to clade 6B.2, and the other 31 strains belonged to clade 6B.1. A/Guangdong/887/2017 (887/2017) and A/Guangdong/752/2017 (752/2017) (clade 6B.1), 184/2016 (clade 6B.2) and A/California/04/2009 (CA04) replicated efficiently in MDCK cells and A549 cells, as well as the turbinates of guinea pigs. 184/2016 and CA04 could transmit among guinea pigs through physical contact.

**Conclusion:**

Our findings provide novel insights into the evolution, pathogenicity, and transmission of pdm09 virus. The results show that enhancing surveillance of pdm09 viruses and timely evaluation of their virulence are essential.

## Introduction

The pandemic of H1N1 influenza (pdm09) broke out in Mexico in March and early April 2009 (Wong et al., 2015). The virus spread to more than 214 countries and regions, causing an estimated 151,700 to 575,400 deaths in the first year of the outbreak alone and generating the first influenza pandemic of the 21st century (Smith et al., 2009; Fineberg, 2014). Pdm09 is a unique combination of influenza viruses in which the neuraminidase (NA) gene and M gene are from swine influenza A viruses circulating in Eurasia, the polymerase PB1 gene is from the H3N2 seasonal influenza virus, the polymerase PA gene and polymerase PB2 gene are from avian influenza A viruses circulating in North America, and the hemagglutinin (HA) gene, nucleoprotein (NP) gene, and non-structural protein (NS) gene are from classic swine influenza A viruses also circulating in North America (Shinde et al., 2009).

Pdm09 viruses have been divided into at least eight genetic groups and several subgroups, among which group 1 contains A/California/07/2009 (Radovanov et al., 2020). Clade 7 is the predominant lineage of pdm09 viruses that were prevalent in Japan, Morocco, and Pakistan in 2009–2010 (Bashir Aamir et al., 2012; Khandaker et al., 2013; El Rhaffouli et al., 2014). Viruses of clades 3 and 6 were common in 2011, while clade 7 viruses became dominant in 2012, and subtype 6B, subtype 6C, and clade 7 were dominant in 2013 in Cuba (Arencibia et al., 2015). The epidemic types of pdm09 were clade 6B.1A and subclades 6B.1A1, 6B.1A5, and 6B.1A6 in 2017–2019 in Thailand. The pdm09 viruses of 6B.1A5A have been circulating in Bulgaria since 2019 (Korsun et al., 2021; Suntronwong et al., 2021).

In China, subtype 6B, subtype 6C, subtype 6A, and clade 7 viruses were prevalent in Dalian in 2010–2014 (Han et al., 2016). From August 2009 to 2017, most viruses belonged to clades 1.7, 6C, 6B.1, and 6B.2 in Yantai (Liu et al., 2019). Subtype 6B.1 was prevalent in 2015–2017 in Beijing (Fang et al., 2015; Liu et al., 2020). The main epidemic lineages were subtype 6B.2 in 2014–2016, subtype 6B.1 in 2017–2018, and subtype 6B.1A in 2018 in Lincang (Zhao et al., 2020). After 2013, the major global epidemic pdm09 viruses belonged to subtype 6B.

Since its outbreak, the pdm09 virus has changed at different molecular sites that affect the replication capacity of the virus in cells *in vitro*. Internationally, the 24-h replication titer is 10–times lower for A/California/04/09 with D222G mutation in the human bronchial epithelial cell line Calu–3 than that of the wild-type virus (Belser et al., 2011). The replication titer of ACU009 and ACU022 with PA protein (V100I, P224S, N321K, I330V, and R362K) and NS1 protein (E55K, L90I, I123V, E125D, K131E, and N205S) is about 10,000 times higher than that of CA/04/09 in human lung adenocarcinoma epithelial (A549) cells (Nogales et al., 2018). In China, the replication titer of A/Sichuan/1/2009 with HA (L32I), PA (A343T), PB1 (K353R and T566A), and PB2 (T471M) in Madin–Darby canine kidney (MDCK) cells at 12 h is significantly higher than that of other strains, such as A/California/04/2009 (Xu et al., 2011). The replication titer is significantly higher for A/Changchun/01/2009 with PB1-T296R mutation derived from passage in mice in MDCK and A549 cells at 36 h than that of the wild type (Yu et al., 2015). Mutation S186P in HA protein could significantly enhance the viral titer of A/Guangdong/GLW/2018 in MDCK cells (Xing et al., 2021).

The A/Beijing/501/2009 H1N1 virus can effectively replicate in the turbinate, trachea, lungs, and brain of ferrets intranasally infected at a dose of 10^7^ TCID_50_. This causes up to 10% body weight loss, as well as apoptosis and severe inflammation in the lungs (Yang et al., 2012). Similarly, A/California/07/2009 effectively replicates in the turbinate, trachea, and lungs of ferrets infected at a dose of 10^6^ TCID_50_, causing more than 20% body weight loss and 27% mortality (Rowe et al., 2010). P224S mutation in PA protein and T588I mutation in PB2 protein enhance the pathogenicity of CA04 in mice (Sun et al., 2014). 627 K and 701N mutations can significantly increase the polymerase activity of CA04 (Song et al., 2011). NA protein E119D mutation can significantly reduce CA04 virus replication and mortality in the lungs of C57BL/6 mice. Compared to the wild type, CA04 with E119D mutation in NA protein shows decreased viral titers in nasal wash, but the ability of physical contact transmission among guinea pigs is still maintained (Abed et al., 2016). Pdm09 viruses continue to circulate and evolve. To better understand their genetic diversity, pathogenicity, and transmission, the aims of this study were (1) to reveal genetic diversity and circulation of pdm09 in China in 2009–2020 and (2) to detect viral replication in mammalian cells, and to assess its viral pathogenicity and transmission in guinea pigs.

## Materials and methods

### Viruses, cells, and Guinea pigs

Thirty-three viruses were isolated and purified in the Guangdong Provincial Center for Disease Control and Prevention in 2016–2017 and were propagated in MDCK cells. MDCK cells and A549 cells were stored in the laboratory. All cells were cultured in Dulbecco’s modified Eagle’s medium (DMEM) (Gibco, Grand Island, NY, USA) containing 10% fetal bovine serum (Gibco, Grand Island, NY, USA) and 1% penicillin–streptomycin (PS) (BI, Kibbutz Beit Haemek, Israel). Twenty-four Hartley–strain guinea pigs were purchased from Liaoning Changsheng Biotechnology Co. Ltd. The guinea pigs were female, 6 weeks old, and specific pathogen free (SPF) (Wu et al., 2021).

### Genomic sequencing and phylogenetic analysis

The total viral RNA was extracted by an RNAfast 200 purification kit (Fastagen Biotech, Shanghai, China). RT-PCR and PCR were conducted according to a previous study (Hoffmann et al., 2001). Sequencing was performed by Tianyi Huiyuan Biotechnology Co. in Guangdong province, China, and spliced using the software SeqMan version 7.1 (Lasergene). The sequences were deposited in the GISAID database with accession numbers 14193573–14193604 and 14193606.

To analyze the phylogenetic evolution of viruses isolated in Guangdong in 2016–2017, the maximum likelihood (ML) phylogenetic tree of the HA gene was constructed. All available genome sequences of Pdm09 viruses were downloaded from the GISAID and GenBank Flu databases in 2009–2020. Sequence alignments were conducted using MAFFT version 7.149 (Katoh and Standley, 2013). The best-fit model was determined by ModelFinder with the AIC criterion of GTR + F + G4 (Kalyaanamoorthy et al., 2017). We used PhyloSuite software (version 1.2.2) to construct the ML tree with the ultrafast bootstrap model and 1,000 bootstraps (Zhang et al., 2020). The phylogenetic tree was visualized in FigTree version 1.4.3.^1^ We used ITOL version 5 to complete the annotation of the evolutionary tree and to adjust it esthetically (Letunic and Bork, 2021).

### Genetic diversity and haplotypes analysis

The genetic distances for viral sequences were calculated with the R seqinr package (Charif and Lobry, 2007), which relies on the multiple sequence alignment results calculated by the R msa package (Bodenhofer et al., 2015). The ML phylogenetic tree was then constructed with the R ape package (Paradis and Schliep, 2019), and the haplotypes were identified with the R haplotypes package.^2^

### Viral replication in mammalian cells

The *in vitro* replication of 887/2017, 752/2017, 184/2016, and CA04 was characterized with MDCK and A549 cells. When the confluence of the cells in 12–well plates was about 90%, MDCK cells were infected with 887/2017, 752/2017, 184/2016, and CA04 at an MOI of 0.001. A549 cells were inoculated with 887/2017, 752/2017, 184/2016, and CA04 at an MOI of 0.01. At 2 h post infection (hpi), inoculants were discarded. Cells were washed with PBS twice and then cultured in Opti-MEM I Reduced Serum Medium (Sigma–Aldrich, St. Louis, MO) with 1.0 μg/ml of TPCK-treated trypsin and 1% penicillin and streptomycin. Supernatants of cells were collected at 12, 24, 36, 48, 60, and 72 hpi and titrated in MDCK cells (Wu et al., 2021).

### Viral replication in Guinea pigs

To detect virus replication in guinea pigs, six guinea pigs were randomly divided into three groups of two guinea pigs each. Under anesthesia, 887/2017, 752/2017, 184/2016, and CA04 were inoculated intranasally at a dose of 10^6^ TCID_50_ in a volume of 300 μl. Guinea pigs were dissected at 3 DPI and nasal turbinates, tracheas, and lungs were collected. Viral titers in the tissues were titrated in MDCK cells (Wu et al., 2021).

### Transmission of viruses among Guinea pigs

To detect virus transmission, nine guinea pigs were randomly divided into three groups of three guinea pigs each. Under anesthesia, each group was inoculated intranasally with 887/2017, 752/2017, 184/2016, and CA04 in a volume of 300 μl at a dose of 10^6^ TCID_50_. At 1 DPI, 3 naive guinea pigs were co-housed with the infected guinea pigs. Nasal washes from guinea pigs were collected at 2, 4, 6, 8 and 10 DPI and titrated in MDCK cells. The sera were collected at 21 DPI for hemagglutination inhibition (HI) titration (Wu et al., 2021).

### Antigenicity relationship of viruses

Serum samples against 887/2017, 752/2017, and 184/2016 were treated with receptor–destroying enzyme (RDE) (Denka Seiken Co., Tokyo, Japan) at 37°C for 18–20 h. The serum samples were then incubated at 56°C for 30 min. The treated serum samples were diluted 10 times with PBS. HI assay with 0.5% turkey erythrocytes (Wu et al., 2021). *R*-values which represent antigenic differences were calculated according to previous studies (Archetti and Horsfall 1950; Zhang et al., 2019).

### Biosafety and animal handling

The laboratory and guinea pig experiments were carried out in compliance with the approved protocols of the biosafety committee of South China Agriculture University. The handling of guinea pigs was performed in accordance with the approved guideline of the experimental animal administration and ethics committee of South China Agriculture University (SCAUABSL2020–007; 26 May 2020).

## Results

### Geographic distribution and genetic diversity of pdm09 in China in 2009–2020

We collected viruses from China in 2009–2020 based on the literature. There were 739, 280, 185, 37, 83, 62, 154, 156, 230, 745, 378, and 84 viruses for the years 2009 to 2020, respectively (Table S1). To analyze the geographical distribution of pdm/09 viruses in China, the provinces were classified into seven regions on the basis of geography: North (Inner Mongolia, Hebei, Shanxi, Tianjin, and Beijing), Northeast (Jilin, Liaoning, and Heilongjiang), Northwest (Shaanxi, Gansu, Qinghai, Ningxia, and Xinjiang), East (Anhui, Jiangxi, Shandong, Fujian, Jiangsu, Shanghai, and Zhejiang), Central (Hubei, Hunan, and Henan), South (Guangdong, Hainan, Hong Kong, Macau, and Guangxi), and Southwest (Chongqing, Sichuan, Guizhou, Tibet, and Yunnan).

**Figure 1:**
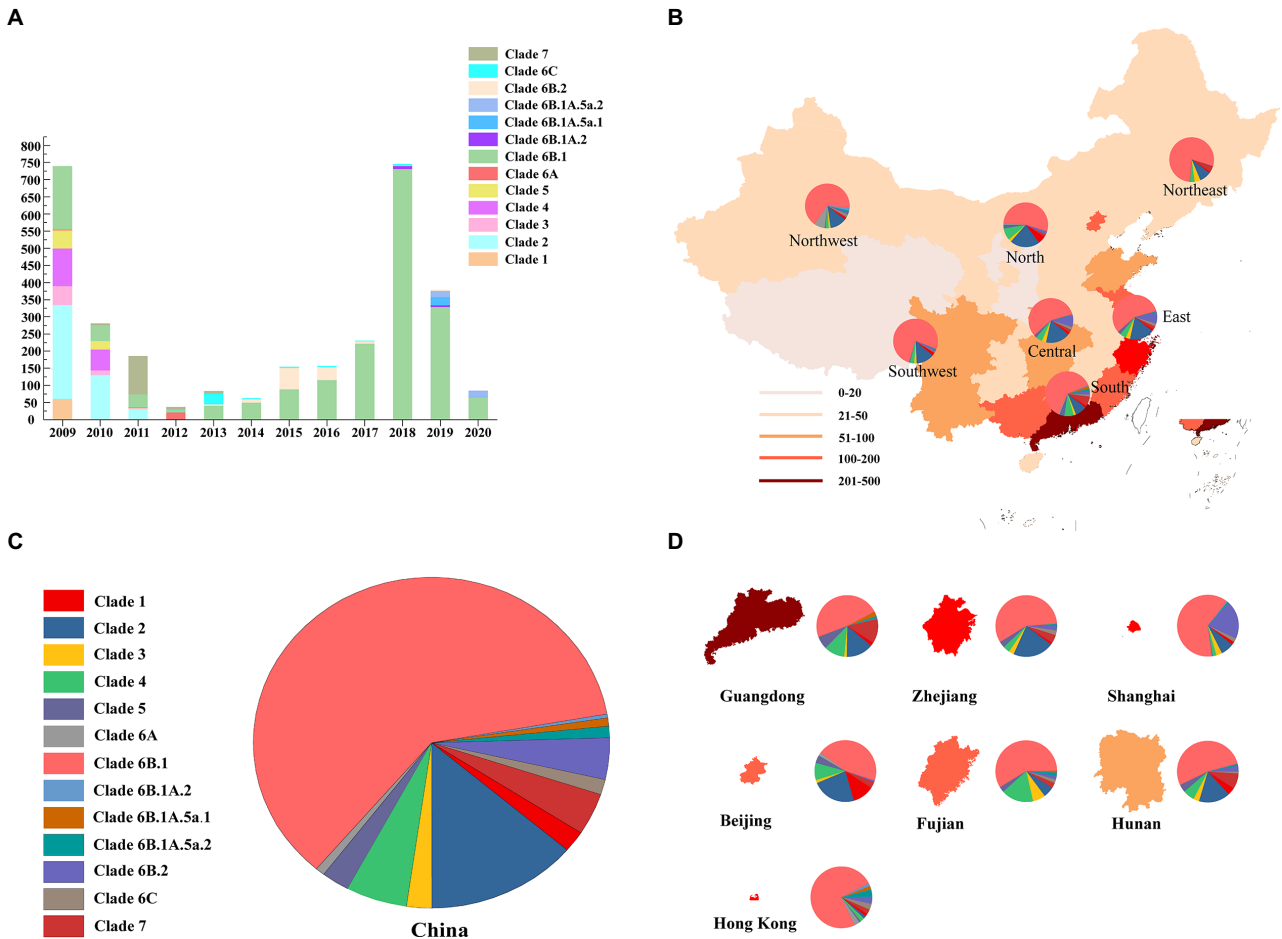
Geographic distribution and genetic diversity of pdm09 viruses in China. **(A)** Diversity of genotypes of pdm09 viruses isolated in China, 2009–2020. **(B)** A map of the pdm09 virus clades distribution; China was divided into seven different regions: North (Inner Mongolia, Hebei, Shanxi, Tianjin, and Beijing), North–East (Jilin, Liaoning, and Heilongjiang), North–west (Shaanxi, Gansu, Qinghai, Ningxia, and Xinjiang), East (Anhui, Jiangxi, Shandong, Fujian, Jiangsu, Shanghai, and Zhejiang), Central (Hubei, Hunan, and Henan), South (Guangdong, Hainan, Hong Kong, Macau, and Guangxi), and Southwest (Chongqing, Sichuan, Guizhou, Tibet, and Yunnan). **(C)** The proportion of HA gene of pdm09 viruses in China, 2009–2020. **(D)** The proportion of HA gene of pdm09 viruses in Guangdong, Zhejiang, Shanghai, Beijing, Fujian, and Hunan provinces as well as Hong Kong, 2009–2020.

The results showed that the HA gene were classified into 13 clades (the number of sequences is in parentheses): clade 1 (64); clade 2 (426); clade 3 (71); clade 4 (172); clade 6A (26); clade 6B.1 (1894); clade 6B.1A.2 (11); clade 6B.1A.5a.1 (25); clade 6B.1A.5a.2 (35); clade 6B.2 (126); clade 6C (46), and clade 7 (127) (Figure 1A). In total, 3,038 pdm09 viruses harbored clade 6B.1 (62% of all pdm09 viruses) and clade 6B.2 (4%). Clade 6B.1 pdm09 viruses are the predominant clade, with proportions of 54.1, 78.9, 57.2, 58.6, 61.7, 76.3, and 66.6% in the North, Northeast, East, Central, South, Southwest, and Northeast regions, respectively (Figures 1B–D). The isolation proportion of clade 6B.1 pdm/09 viruses was 57.1%, 74.3%, 96.1%, 98.2%, 86.7%, and 78.5% in 2015–2020, respectively.

### Genetic diversity of eight genes of viruses isolated in the laboratory

**Figure 2:**
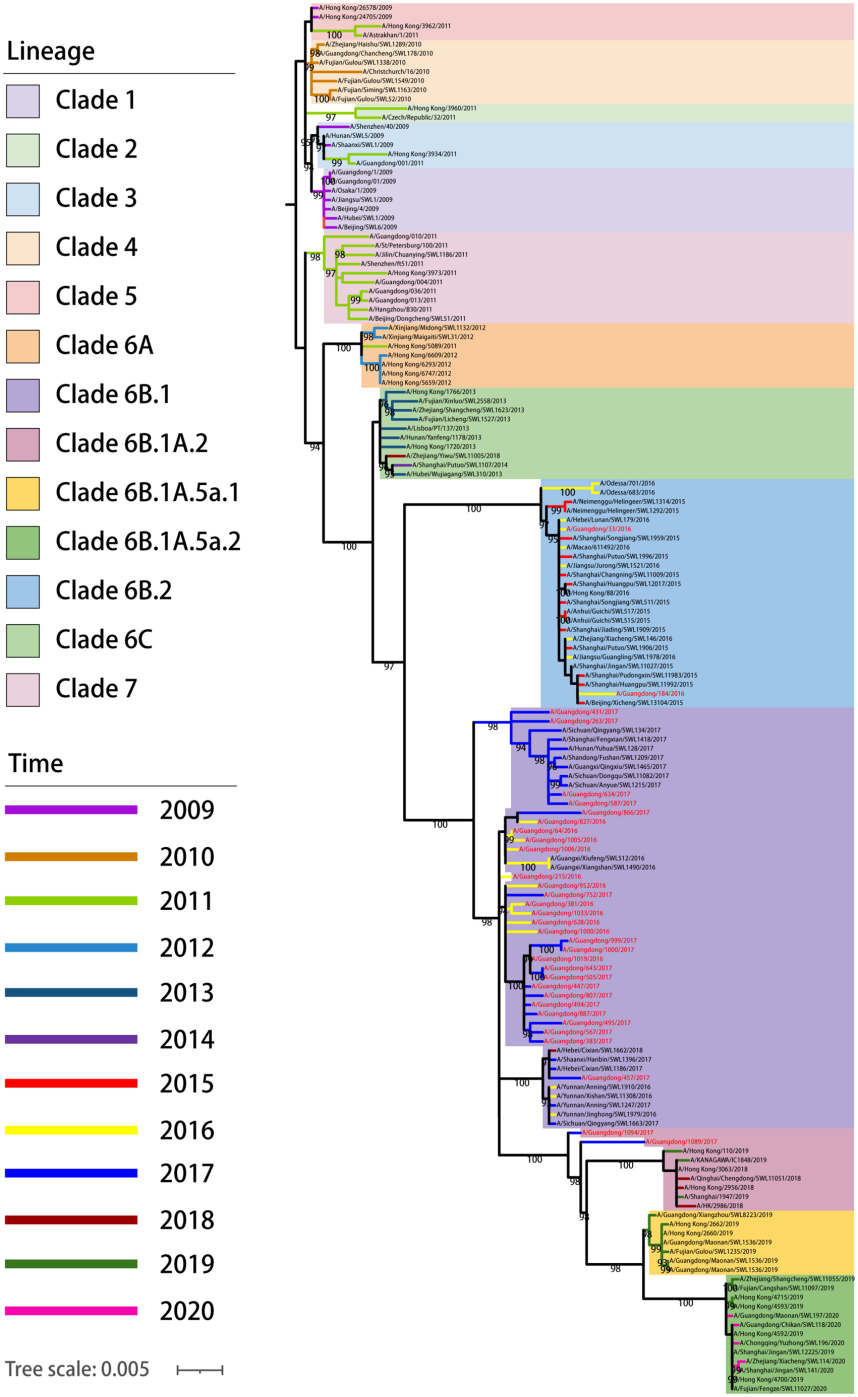
Phylogenetic relationship of HA gene of pdm/09 viruses in China from 2009 to 2020. Different clades and years are denoted by different markers. All branch lengths are scaled according to the numbers of substitutions per site (subs/site). Phylogenetic tree was estimated using genetic distances calculated by maximum likelihood under the GTR + F + G4 model. Pdm09 viruses isolated in this study are marked in red; sequences of viruses with names in black were downloaded from databases.

To reveal the phylogenetic relationship of viruses in Guangdong in 2016–2017, an ML phylogenetic tree was constructed for the HA gene of viruses propagated (Figure 2). The results showed that all 33 strains of the 2009 pandemic H1N1 viruses belonged to clade 6B, among which A/Guangdong/33/2016 and A/Guangdong/184/2016 belonged to subclade 6B.2, and the other 31 strains belonged to subclade 6B.1. We also found that A/Guangdong/1089/2017 and A/Guangdong/1094/2017 belonged to subclade 6B.1.A.2 and was more genetically distant than the other strains, suggesting that the 2009 pandemic H1N1 virus is constantly undergoing adaptive evolution in humans (Figure 2).

### Genetic diversity of viruses in China and North America from 2009 to 2018

**Figure 3:**
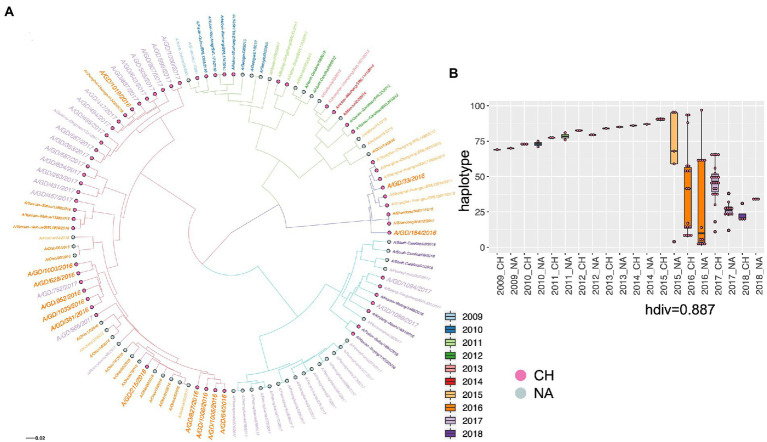
The phylogenetic relationship of H1N1 viruses in Guangdong in 2016 and 2017 with H1N1 viruses in China and North American from 2009 to 2018 was displayed by the ML three of HA gene **(A)** and the haplotype distribution **(B)**. The viruses from different country and different year are shown by diverse colors, and H1N1 viruses in Guangdong in 2016 and 2017 were highlighted by larger size of text. Haplotype: haploid genotype, is a group of alleles in an organism that are inherited together from a single parent. CH: China, NA: North America. hdiv: haplotype diversity.

We introduced viruses that came from the entire region of North America from 2009 to 2018 to see the phylogenetic position of our viruses from a broader view. The results showed that our viruses from 2016 and 2017 share a common parent with the viruses from 2016, which was also addressed by the Florida virus analysis. Importantly, the distribution of haplotypes indicates clear divergence in the year 2015 (Figures 3A,B).

### Multi–mutations in 33 pdm/09 viruses in Guangdong

Molecular characteristic analysis showed a series of functional mutations in PB2, PB1, PA, PA–X, HA, NP, NA, M2, and NS1 proteins (Tables S2 and S3). These proteins are associated with the infectivity, replication, transmission, antigenicity, receptor binding feature, and drug resistance of pdm09 viruses.

### Four viruses effectively replicated in MDCK and A549 cells

**Figure 4:**
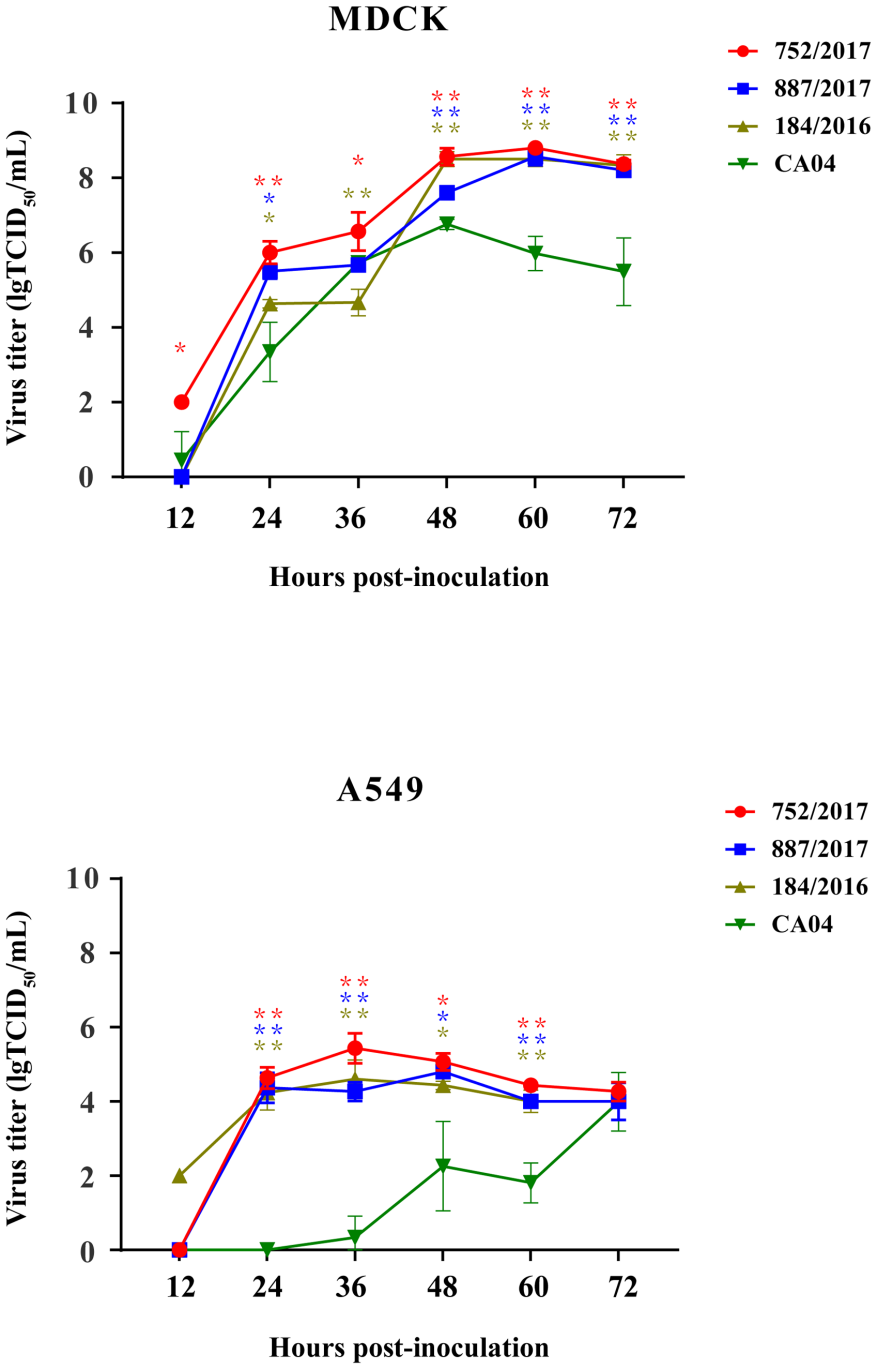
Replication kinetics of 887/2017, 752/2017, 184/2016, and CA04 viruses cultured in MDCK cells with MOI 0.001 and A549 cells with MOI 0.01. The data at each time point represent the mean standard deviation of the three independent experiments. The “*” represents significance of difference, **p < 0.01, *p < 0.05 (Multiple T-test).

To detect the replication of subclades of viruses *in vitro*, 887/2017, 752/2017, and 184/2016 were selected, the ancestor virus CA04 was selected as the control, and their multi–step growth curves were obtained using MDCK and A549 cells. The three viruses exhibited higher replication titers in MDCK cells than A549 cells. In MDCK cells, 887/2017, 752/2017, and 184/2016 reached viral peaks with titers of 8.7, 8.7, and 9.0 lgTCID_50_/mL at 60 hpi, respectively. Compared to CA04, they showed significant differences in replication titers in MDCK cells at 48, 60, and 72 h. In A549 cells, 887/2017 reached a viral peak titer of 4.8 lgTCID_50_/mL at 48 hpi, while 752/2017 and 184/2016 reached viral peaks with titers of 5.8 and 5.2 lgTCID_50_/mL at 36 hpi, respectively. Compared to CA04, they showed significant differences in replication titers in A549 cells at 24, 36, and 60 h compared to CA04 (Figure 4).

### Four viruses replicated efficiently in the turbinates of Guinea pigs

**Figure 5:**
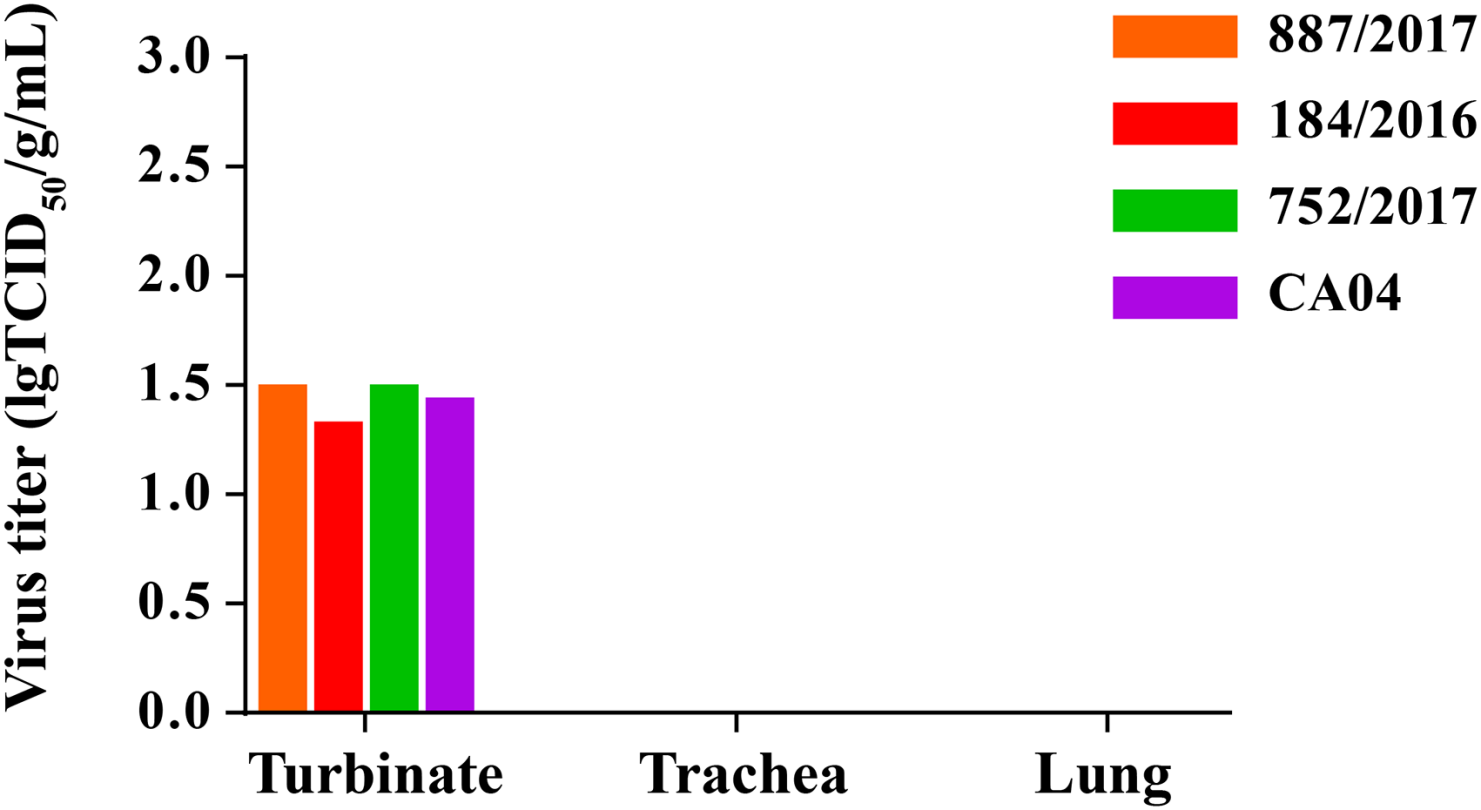
Replication of 887/2017, 752/2017, 184/2016 and CA04 viruses in guinea pigs. Eight female, 6-week-old, SPF, Hartley strain guinea pigs were randomly divided into four groups and each group includes 2 guinea pigs. Groups of guinea pigs were intranasally inoculated with 887/2017, 752/2017, 184/2016, and CA04 at a dose of 10^6^ TCID_50_ in 300 μL under anesthesia, respectively. Guinea pigs were necropsied at 3 DPI, and turbinate, trachea, and lung were collected. The viral titers in tissues of guinea pigs were titrated in MDCK cells.

To detect the replication of 887/2017, 752/2017, 184/2016, and CA04 *in vivo*, 12 guinea pigs were inoculated intranasally with these four viruses at a dose of 10^6^ TCID_50_ in a 300-μL volume. At 3 DPI, the guinea pigs were necropsied, and the turbinates, tracheas, and lungs were collected. Viral titers were then detected in MDCK cells. The viral titers of 887/2017, 752/2017, 184/2016, and CA04 in the turbinate were 1.50, 1.50, 1.40, and 1.44 lgTCID_50_/g/mL, respectively (Figure 5). No virus was detected in the tracheas or lungs of guinea pigs. These results indicated that 887/2017, 752/2017, 184/2016, and CA04 replicated efficiently in the turbinates of guinea pigs without prior adaptation.

### Transmission of A/184/2016 and CA04 virus among Guinea pigs through direct contact

To detect the transmission of 887/2017, 752/2017, 184/2016, and CA04, 12 guinea pigs were randomly divided into three groups of three guinea pigs. Each group was inoculated intranasally with a corresponding virus at a 300-μL dose of 10^6^ TCID_50_. At 1 DPI, three naive guinea pigs were co-caged with the infected guinea pigs. Nasal washes were collected at 2, 4, 6, 8, and 10 DPI, and viral titers were detected in MDCK cells. Guinea pigs were necropsied, and sera were collected at 21 DPI, and HI titers were detected by HI assays.

**Figure 6:**
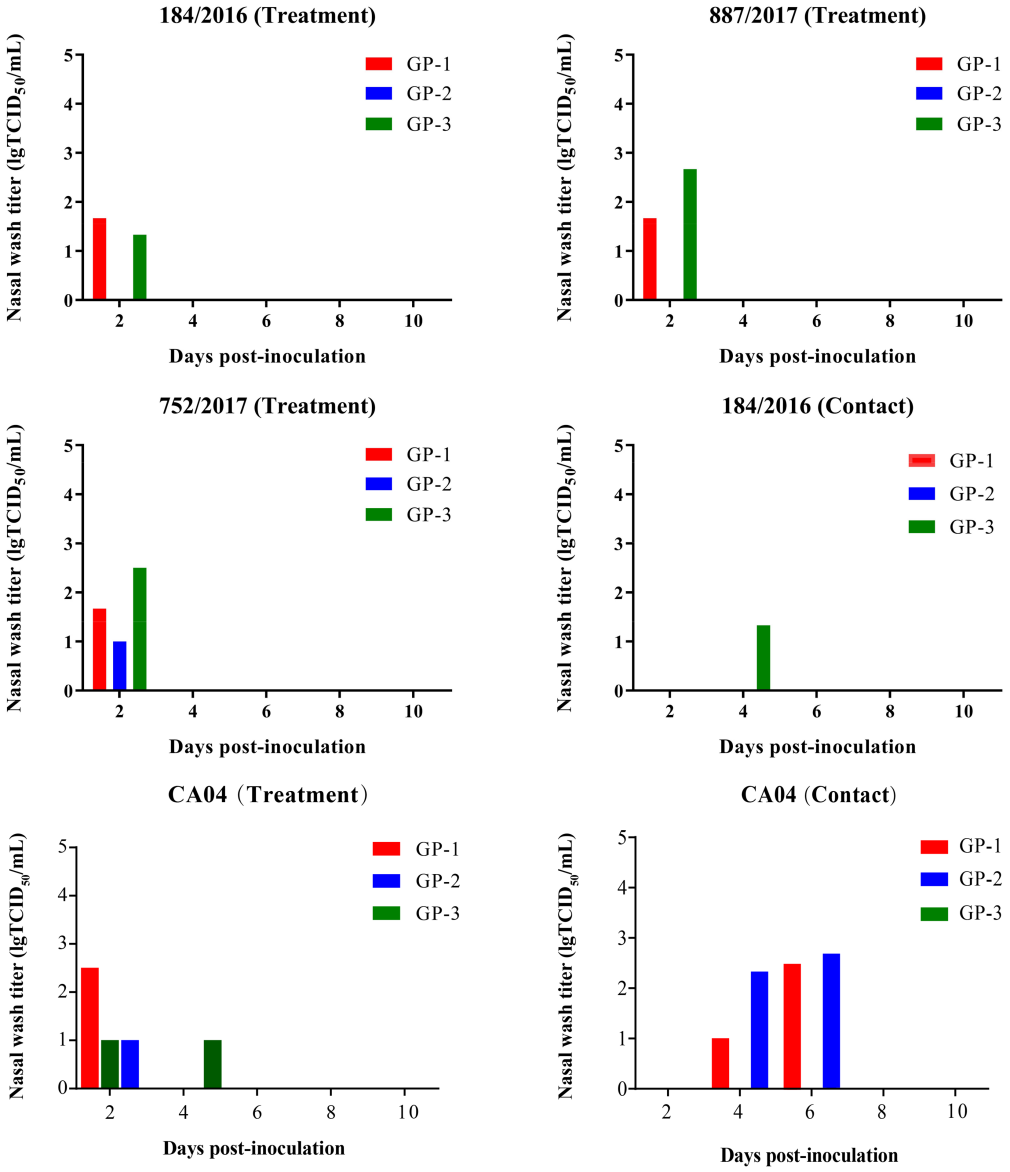
Transmission of 887/2017, 752/2017, 184/2016, and CA04 viruses among guinea pigs. Twelve female, 6-week–old, SPF Hartley strain guinea pigs were randomly divided into three groups and each group includes three guinea pigs. Groups of guinea pigs were intranasally inoculated with 887/2017, 752/2017, 184/2016, and CA04 at a dose of 10^6^ TCID_50_ in 300 μL under anesthesia, respectively. At 1 DPI, three naïve guinea pigs were co-housed with three infected guinea pigs in the same cage. The nasal washes of guinea pigs were collected at 2, 4, 6, 8, and 10 DPI and were titrated in MDCK cells. The sera were collected at 21 DPI for HI titration.

At 2 DPI, 2 of the 3 guinea pigs in the 184/2016 and 887/2017 treatment groups were detected to shed viruses with titers of 1.2–2.9 lgTCID_50_/mL. At 2 DPI, all 3 guinea pigs in the 752/2017 and CA04 treatment group were detected to shed the virus with titers of 1–2.5 lgTCID_50_/mL. At 4 DPI, 1 guinea pig in the 184/2016 contact group was detected to shed the virus with a titer of 1.33 lgTCID_50_/Ml. Two guinea pigs in the CA04 contact group were detected to shed the virus titer of 1.0–2.67 lgTCID_50_/mL at 4 and 6 DPI (Figure 6). At 4 DPI, 1 guinea pig in the 752/2017 treatment group died, and the viral titers in the turbinate, trachea, and lung of the dead guinea pig were 1, 1.33, and 0.97 lgTCID_50_/mL, respectively (Supplementary Figure S1). At 3 and 4DPI, 1 guinea pig in the CA04 treatment group died, respectively.

The HI titers were 1:160–1:320 in the 887/2017 treatment group, 1:80–1:254 in the 752/2017 treatment group, and 1:1,280–1:2,032 in the 184/2016 treatment group. In the 184/2016 contact group, the serum HI titer of the guinea pig that was detected to shed the virus at 4 DPI was 1:508 (Table 1). These findings indicated that 184/2016 could be transmitted efficiently among guinea pigs through direct contact.

**Table 1:** HI titers of sera collected from guinea pigs.

Virus	Treatment guinea pigs			Contact guinea pigs		
	GP1^a^	GP2	GP3	GP1	GP2	GP3
887/2017	320	160	320	<10	<10	<10
752/2017	80	—^b^	254	<10	<10	<10
184/2016	1,280	1,280	2032	<10	<10	508
PBS	<10	<10	<10	<10	<10	<10

a GP means guinea pig. HI titers were expressed at geometric mean titer.

b means the GP2 of 752/2017 dead at 4dpi, and no serum was available.

### Antigenicity relationship of viruses

Guinea pig serum samples against 887/2017, 752/2017, and 184/2016 had agglutination inhibition titers for 887/2017, 752/2017, and 184/2016 strains. The agglutination titer was the highest for the 184/2016 strain (640, 1,016, and 1,280, respectively.). The antigenicity showed significant difference between 752/2017 and 184/2016 (Table 2).

**Table 2:** Antigenic relatedness (*R*-value) among the three viruses as determined by the degree of cross-HI activity.

	*R*-value			
	752/2017	887/2017		184/2016
752/2017	1	1		**0.5**
887/2017	1	1		0.891
184/2016	**0.5**	0.891		1

*R*-values shown in bold indicate significant differences.

*R* = 1 indicates the two viruses pose the same antigenicity, 0.67 ≤ *R* < 1 indicates no significant antigenic difference between the two viruses, and *R* < 0.5 indicates a significant antigenic difference between the viruses.

## Discussion

Here, we systematically described the overall picture of the geographic distribution and genetic diversity of pdm09 viruses, and found that the clade 6B.1 pdm/09 viruses were predominant in China in 2014–2020. To characterize pdm09 viruses in China after 2015, 33 viruses isolated in Guangdong in 2016–2017 were analyzed. We found that pdm09 viruses of clades 6B.2 and 6B.1 acquired a series of mutations related to viral pathogenicity and transmission and co-circulated in Guangdong, and found that compared to CA04, 887/2017 and 752/2017 (clade 6B.1) and 184/2016 (clade 6B.2) replicated efficiently in MDCK cells and A549 cells at different times, and that 184/2016 and CA04 could transmit among guinea pigs through physical contact. Finally, we found that a clear differentiation time point appeared in 2015, before which the evolution trend of pdm09 viruses in China was similar to that in North America but then showed a different trend after that point.

Compared to pdm09 viruses from the same period, viruses circulating in Guangdong showed different genetic characteristics. Viruses circulating in Guangdong in 2016–2017 had the same evolutionary parents as the viruses from China and North America in 2016, while 33/2016 and 184/2016 shared evolutionary parents with the 2015 viruses. Notably, there was a clear differentiation of the viruses in 2015. This differentiation may be due to the different evolutionary sources of these viruses and those viruses produced in different years.

Mutations at key amino acid sites in some proteins could change viral replication, pathogenicity, and drug resistance. Double mutations of V106I and N248D in NA protein can enhance viral replication in the human body. The substitutions K353R and T566A in PB1 proteins and T471M in PB2 proteins have been shown to be potential key virulence factors (Xu et al., 2011). The mutations V100I in NP protein and I123V in NS1 protein play a role in pdm09 adaptation to human hosts and increased virulence (Pan et al., 2010). H275Y and E119D mutation or E119D and H274Y double mutations in NA protein can cause pdm09 to have oseltamivir resistance (Collins et al., 2009; Wang et al., 2010; Hanpaibool et al., 2020). Double mutations of S31N and V27A in M2 protein can lead to amantadine drugs (Antón et al., 2010).

All 33 viruses in this study had N248D and H274Y mutations in NA protein, V100I in NP protein, I123V in NS1 protein, and K353R in PB1 protein. Only A/Guangdong/1094/2017 had S31N and V27A mutations in M2 protein. Further experiments are needed to validate whether these mutations affect the replication, virulence, and drug resistance of viruses.

Recombinant viruses reached peak titers of 6–7 lgTCID_50_/mL at 36–48 hpi in MDCK cells (Tu et al., 2018), while three of our viruses peaked at 60 hpi with 8.7–9.0 lgTCID_50_/mL in MDCK cells at 0.001 MOI. Compared to CA04, these three viruses had significantly enhanced replication ability in A549 cells and MDCK cells. This discrepancy may be caused by the cell culture conditions, inoculum dose, and characteristics of the viruses. The choice of MOI could affect viral infection and spread in these cells (Xia et al., 2017). Furthermore, T296R substitution in PB1 protein could enhance polymerase activity of pdm09 virus in MDCK cells (Yu et al., 2015). This substitution also occurred in 887/2017, 752/2017, and 184/2016. This replacement may also account for the high replication of these three viruses in MDCK cells.

The viral titers were about 10,000–times higher for ACU009 and ACU022 with V100I, P224S, N321K, I330V, and R362K in PA proteins and E55K, L90I, I123V, E125D, K131E, and N205S in NS1 proteins in A549 cells than that of CA/04/09 (Nogales et al., 2018). These substitutions in PA protein and V100I, P224S, R221Q, and L229S in PA–X protein are likely beneficial for the viruses as they have become fixed at the global level (Nogales et al., 2018). The mutations in NS1 protein gave the viruses the ability to suppress host gene expression (Clark et al., 2017). Notably, similar amino acid substitutions were found in PA and NS1 proteins of strains 887/2017, 752/2017, and 184/2016, but not for 184/2016, which lacks E125D in the NS1 protein.

The PA and PA–X proteins of 887/2017 and 184/2016 also had these amino acid changes. In addition, the viral titer was significantly higher for A/Changchun/01/2009 with T296R in PB1 protein in A549 cells at 36 h than that of the parental strain (Yu et al., 2015). The same amino acid substitution was also found in the PB1 protein of the three viruses. Taken together, all these mutations may be associated with high replication of 887/2017, 752/2017, and 184/2016 in A549 cells.

V100I in NP protein and I123V in NS1 protein are involved in human adaption and virulence of pdm09 viruses (Pan et al., 2010). Notably, 887/2017, 752/2017, and 184/2016 show the same mutations, which may account for the efficient replication of the viruses in guinea pigs. The HA gene plays an important role in the effectiveness of the direct transmission of CA04 between guinea pigs (He et al., 2014). PA and NS genes of SC/09(H1N1) can make the hybrid DK/35(H5N1) effectively spread among guinea pigs through respiratory droplets (Zhang et al., 2013). P224S in PA protein, T588I in PB2, V106I in NA, and I123V in NS contribute to the lethal phenotype of A/swine/Shandong/731/2009 in mice (Sun et al., 2014). Multiple mutations of V169T, A278S, E508G, and D518E in HA protein and V67I in NA protein are associated with sore throat after illness in humans (Liu et al., 2020), and only 33/2016 and 184/2016 show these mutations. The 184/2016 strain can be effectively transmitted among guinea pigs through direct contact. More experiments are needed to confirm whether the difference is related to these proteins and mutations. In conclusion, pdm09 viruses are evolving and becoming more genetically diverse, and their replication, pathogenicity, and antigenicity continue to vary.

## Conclusion

Our results showed that the HA gene of pdm09 virus were classified into 13 clades, among which Clade 6B.1 pdm09 viruses have been predominantly circulation in China. Clade 6B.1 and clade 6B.2 pdm09 viruses replicated efficiently in mammalian cells and guinea pigs, of which clade 6B.2 virus exhibited contact transmission ability. These data can improve our understanding of pdm09’s evolution, replication, and transmission characteristics. As evolution continues, comprehensive surveillance should be taken to control and prevent the spread of pdm09 virus.

## Data availability statement

The datasets presented in this study can be found in online repositories. The names of the repository/repositories and accession number(s) can be found in the article/Supplementary material.

## Ethics statement

The animal study was reviewed and approved by South China Agriculture University (SCAUABSL2020–7; 13 March 2021) approved guideline.

## Author contributions

ML, HS, and CK: design of the work. YW, HL: the acquisition analysis. HL, MH, and XQ: interpretation of data. ZP, XC, YL, and RZ: the creation of new software used in the work. HS, ML, HL have drafted the work or substantively revised it. All authors contributed to the article and approved the submitted version.

## Funding

This work was supported by special fund for scientific innovation strategy–construction of high-level Academy of Agriculture Science–Distinguished Scholar (R2020PY–JC001).

## Conflict of interest

The authors declare that the research was conducted in the absence of any commercial or financial relationships that could be construed as a potential conflict of interest.

## Publisher’s note

All claims expressed in this article are solely those of the authors and do not necessarily represent those of their affiliated organizations, or those of the publisher, the editors and the reviewers. Any product that may be evaluated in this article, or claim that may be made by its manufacturer, is not guaranteed or endorsed by the publisher.

## Footnotes

^1^ http://tree.bio.ed.ac.uk/software/figtree/

^2^ https://cran.r-project.org/web/packages/haplotypes/index.html

## Supplementary material

SUPPLEMENTARY FIGURE S1 Figure 1 Viral titers in tissues of dead guinea pigs in the 752/2017 treatment group At 4 DPI, viral titers in turbinate, trachea, and lung of dead guinea pigs in the 752/2017 treatment group.
