# Supplementary figures and images for: The genetic diversity, replication, and transmission of 2009 pandemic H1N1 viruses in China
Source: Front Microbiol. 2023 Feb 17;14:1110100. doi: 10.3389/fmicb.2023.1110100 (PMC9982095; doi:10.3389/fmicb.2023.1110100)

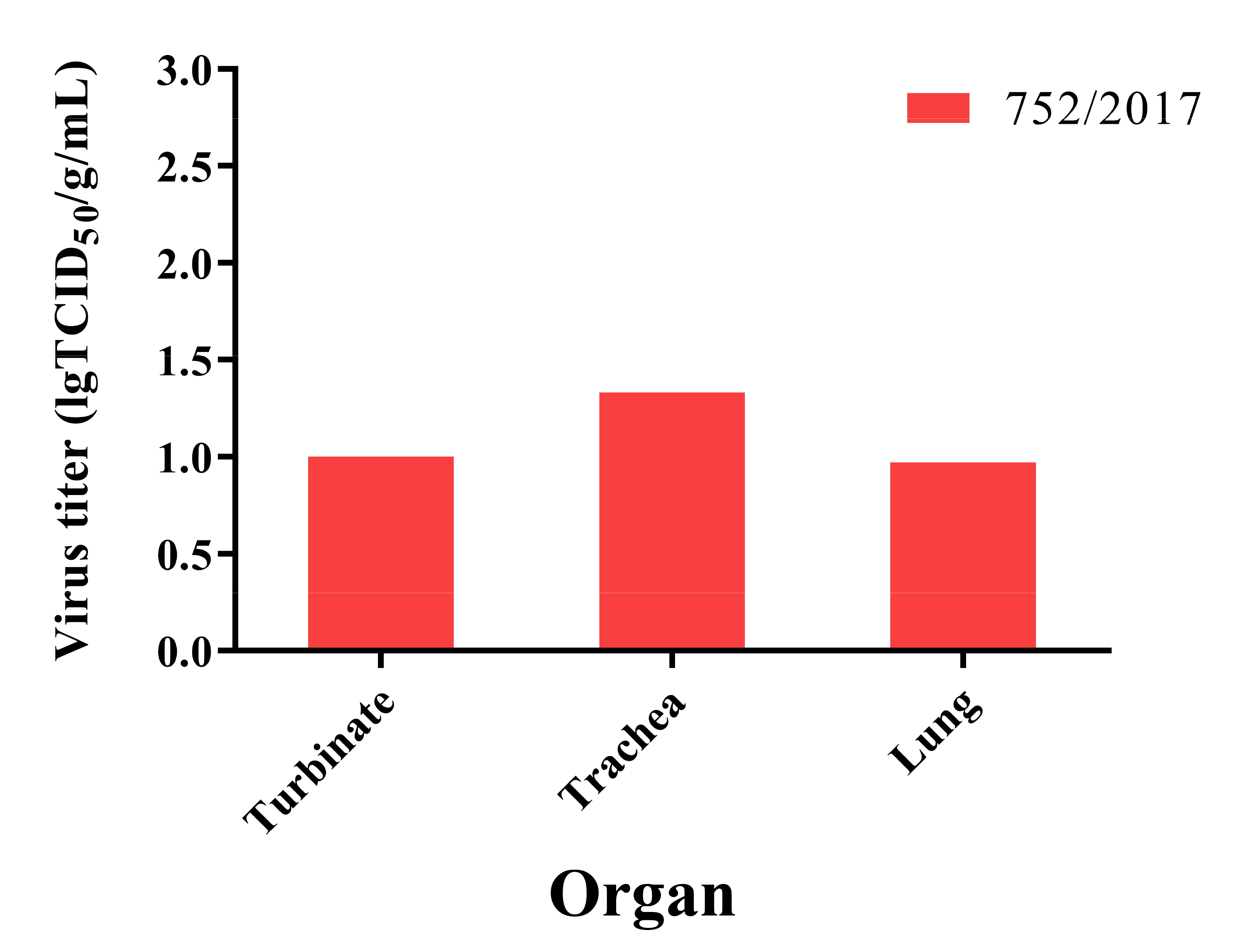

Supplement: SUPPLEMENTARY FIGURE S1 — Figure 1 Viral titers in tissues of dead guinea pigs in the 752/2017 treatment group At 4 DPI, viral titers in turbinate, trachea, and lung of dead guinea pigs in the 752/2017 treatment group. [file Image_1.tif]
