# Supplementary material for: The genetic diversity, replication, and transmission of 2009 pandemic H1N1 viruses in China
Source: Front Microbiol. 2023 Feb 17;14:1110100. doi: 10.3389/fmicb.2023.1110100 (PMC9982095; doi:10.3389/fmicb.2023.1110100)
Supplement: Supplementary file 3 [file Table_2.docx]

| **Strains**  **(2016)** | **Gene** | | | | | | | | | | | | | | | | | | | | | | | | | | | | | | | | | | | | | | | | | | | | | | | | | | | | | | |
| --- | --- | --- | --- | --- | --- | --- | --- | --- | --- | --- | --- | --- | --- | --- | --- | --- | --- | --- | --- | --- | --- | --- | --- | --- | --- | --- | --- | --- | --- | --- | --- | --- | --- | --- | --- | --- | --- | --- | --- | --- | --- | --- | --- | --- | --- | --- | --- | --- | --- | --- | --- | --- | --- | --- | --- |
|  | **PA** | | | | | |  | **PA-X** | | |  | | **PB1** | |  | | **PB2** | | | **M2** | | | |  | | **NA** | | | | | | |  | | | **NS1** | | | | | | **NP** | | | |  | | | **HA** | | | | | |  |
|  | **343** | **224** | **100** | **321** | **330** | **362** |  | **204** | **221** | **229** |  | **296** | **353** | **566** |  | **588** | **158** | **471** |  | | **31** | **27** |  | **119** | **264** | | **67** | **275** | **106** | **248** | **274** |  | | **55** | **90** | | **123** | **125** | **131** | **205** |  | | **100** |  | **169** | | **278** | **508** | | **222** | **518** | **159** | **158** |  |  |
|  | **A** | **P** | **V** | **N** | **I** | **R** |  | **N** | **R** | **L** |  | **T** | **K** | **T** |  | **T** | **E** | **T** |  | | **S** | **V** |  | **E** | **V** | | **V** | **H** | **V** | **N** | **H** |  | | **E** | **L** | | **I** | **E** | **K** | **N** |  | | **V** |  | **V** | | **A** | **E** | | **D** | **D** | **N** | **G** |  |  |
| **33** | **A** | **S** | **I** | **K** | **V** | **K** |  | **N** | **Q** | **S** |  | **R** | **R** | **T** |  | **T** | **E** | **T** |  | | **N** | **V** |  | **E** | **I** | | **I** | **H** | **V** | **D** | **Y** |  | | **K** | **I** | | **V** | **E** | **E** | **S** |  | | **I** |  | **T** | | **S** | **G** | | **R** | **E** | **K** | **A** |  |  |
| **64** | **A** | **S** | **I** | **K** | **V** | **K** |  | **S** | **Q** | **S** |  | **R** | **R** | **T** |  | **T** | **E** | **T** |  | | **N** | **V** |  | **E** | **I** | | **V** | **H** | **V** | **D** | **Y** |  | | **K** | **I** | | **V** | **D** | **E** | **S** |  | | **I** |  | **V** | | **A** | **E** | | **R** | **D** | **K** | **A** |  |  |
| **184** | **A** | **S** | **I** | **K** | **V** | **K** |  | **S** | **Q** | **S** |  | **R** | **R** | **T** |  | **T** | **E** | **T** |  | | **N** | **V** |  | **E** | **I** | | **I** | **H** | **V** | **D** | **Y** |  | | **K** | **I** | | **V** | **E** | **E** | **S** |  | | **I** |  | **T** | | **S** | **G** | | **R** | **E** | **K** | **A** |  |  |
| **215** | **A** | **S** | **I** | **K** | **V** | **K** |  | **S** | **Q** | **S** |  | **R** | **R** | **T** |  | **T** | **E** | **T** |  | | **N** | **V** |  | **E** | **I** | | **V** | **H** | **V** | **D** | **Y** |  | | **K** | **I** | | **V** | **D** | **E** | **S** |  | | **I** |  | **V** | | **A** | **E** | | **R** | **D** | **K** | **A** |  |  |
| **381** | **A** | **S** | **I** | **K** | **V** | **K** |  | **S** | **Q** | **S** |  | **R** | **R** | **T** |  | **T** | **E** | **T** |  | | **N** | **V** |  | **E** | **I** | | **V** | **H** | **V** | **D** | **Y** |  | | **K** | **I** | | **V** | **N** | **E** | **S** |  | | **I** |  | **V** | | **A** | **E** | | **R** | **D** | **K** | **A** |  |  |
| **628** | **A** | **S** | **I** | **K** | **V** | **K** |  | **S** | **Q** | **S** |  | **R** | **R** | **T** |  | **T** | **E** | **T** |  | | **N** | **V** |  | **E** | **I** | | **V** | **H** | **V** | **D** | **Y** |  | | **K** | **I** | | **V** | **D** | **E** | **S** |  | | **I** |  | **V** | | **A** | **E** | | **R** | **D** | **K** | **A** |  |  |
| **827** | **A** | **S** | **I** | **K** | **V** | **K** |  | **S** | **Q** | **S** |  | **R** | **R** | **T** |  | **T** | **E** | **T** |  | | **N** | **V** |  | **E** | **I** | | **V** | **H** | **V** | **G** | **Y** |  | | **K** | **I** | | **V** | **D** | **E** | **S** |  | | **I** |  | **V** | | **A** | **E** | | **R** | **D** | **K** | **A** |  |  |
| **952** | **A** | **S** | **I** | **K** | **V** | **K** |  | **S** | **Q** | **S** |  | **R** | **R** | **T** |  | **T** | **E** | **T** |  | | **N** | **V** |  | **E** | **I** | | **V** | **H** | **V** | **D** | **Y** |  | | **K** | **I** | | **V** | **D** | **E** | **S** |  | | **I** |  | **V** | | **A** | **E** | | **R** | **D** | **K** | **A** |  |  |
| **1000** | **A** | **S** | **I** | **K** | **V** | **K** |  | **S** | **Q** | **S** |  | **R** | **R** | **T** |  | **T** | **E** | **T** |  | | **N** | **V** |  | **E** | **I** | | **V** | **H** | **V** | **D** | **Y** |  | | **K** | **I** | | **V** | **D** | **E** | **S** |  | | **I** |  | **V** | | **A** | **E** | | **R** | **D** | **K** | **A** |  |  |
| **1005** | **A** | **S** | **I** | **K** | **V** | **K** |  | **S** | **Q** | **S** |  | **R** | **R** | **T** |  | **T** | **E** | **T** |  | | **N** | **V** |  | **E** | **I** | | **V** | **H** | **V** | **D** | **Y** |  | | **K** | **I** | | **V** | **D** | **E** | **S** |  | | **I** |  | **V** | | **A** | **E** | | **R** | **D** | **K** | **A** |  |  |
| **1006** | **A** | **S** | **I** | **K** | **V** | **K** |  | **S** | **Q** | **S** |  | **R** | **R** | **T** |  | **T** | **E** | **T** |  | | **N** | **V** |  | **E** | **I** | | **V** | **H** | **V** | **D** | **Y** |  | | **K** | **I** | | **V** | **D** | **E** | **S** |  | | **I** |  | **V** | | **A** | **E** | | **R** | **D** | **K** | **A** |  |  |
| **1019** | **A** | **S** | **I** | **K** | **V** | **K** |  | **S** | **Q** | **S** |  | **R** | **R** | **T** |  | **T** | **E** | **T** |  | | **N** | **V** |  | **E** | **I** | | **V** | **H** | **V** | **D** | **Y** |  | | **K** | **I** | | **V** | **D** | **E** | **S** |  | | **I** |  | **V** | | **A** | **E** | | **R** | **D** | **K** | **A** |  |  |
| **1033** | **A** | **S** | **I** | **K** | **V** | **K** |  | **S** | **Q** | **S** |  | **R** | **R** | **T** |  | **T** | **E** | **T** |  | | **N** | **V** |  | **E** | **T** | | **V** | **H** | **V** | **D** | **Y** |  | | **K** | **I** | | **V** | **D** | **E** | **S** |  | | **I** |  | **V** | | **A** | **E** | | **R** | **D** | **K** | **A** |  |  |

**Supplementary table 2.** Mutations of pdm09 viruses isolated in Guangdong province in 2016.
